# Suppression of Nodule Formation by RNAi Knock-Down of *Bax inhibitor-1a* in *Lotus japonicus*

**DOI:** 10.3390/genes16010058

**Published:** 2025-01-06

**Authors:** Fuxiao Jin, Danxia Ke, Lu Lu, Qianqian Hu, Chanjuan Zhang, Chao Li, Wanwan Liang, Songli Yuan, Haifeng Chen

**Affiliations:** 1 Key Laboratory of Biology and Genetic Improvement of Oil Crops, Ministry of Agriculture and Rural Affairs, Oil Crops Research Institute of Chinese Academy of Agricultural Sciences, Wuhan 430062, China; jinfuxiao0610@163.com (F.J.); jdufnfm@163.com (L.L.); m13477402361@163.com (Q.H.); zhangchanjuan@caas.cn (C.Z.); lichao06@caas.cn (C.L.); wanwanliang2024@163.com (W.L.); chenhaifeng@caas.cn (H.C.); 2 College of Life Sciences & Institute for Conservation and Utilization of Agro-Bioresources in Dabie Mountains, Xinyang Normal University, Xinyang 464031, China; kdx_029@163.com

**Keywords:** Bax inhibitor-1, root nodule symbiosis, RNA interference, nodulation, infection thread, nodule formation

## Abstract

Background/Objectives: The balanced regulation of innate immunity plays essential roles in rhizobial infection and the establishment and maintenance of symbiosis. The evolutionarily conserved cell death suppressor Bax inhibitor-1 plays dual roles in nodule symbiosis, providing a valuable clue in balancing immunity and symbiosis, while it remains largely unexplored in the legume *Lotus japonicus*. Methods/Results: In the present report, the *BI-1* gene family of *L. japonicus* was identified and characterized. We identified 6 *BI-1* genes that translate into peptides containing 240–255 amino acids with different structural characteristics and isoelectric points. We performed phylogenetic analyses and detected evolutionary conservation and divergence among BI-1 proteins from *L. japonicus*, *Glycine max*, *Medicago truncatula*, *Arabidopsis thaliana*, and *Oryza sativa*. Expression profiles among different roots indicated that the inoculation of MAFF303099 significantly increased the expression of most of the *L. japonicus* BI-1 family genes. We down-regulated the transcripts of *LjBI-1a* by RNA interference and observed that *LjBI-1a* promotes nodulation and nodule formation. Conclusions: These discoveries shed light on the functions of *BI-1* genes in *L. japonicus*, and simultaneously emphasize the potential application of *LjBI-1a* in enhancing the symbiotic nitrogen fixation ability of legumes.

## 1. Introduction

In agricultural production, nitrogen is a core element determining biomass and yield [1], while excessive use of nitrogen fertilizers not only raises production expenses but also leads to soil and water pollution, causing adverse environmental impacts [2,3]. Symbiotic nitrogen fixation (SNF) is capable of transforming atmospheric nitrogen into ammonia that can be absorbed and utilized by plants and subsequently participate in the material cycle of ecosystems [4,5], thereby reducing dependence on industrial nitrogen fertilizers and minimizing the environmental impact of agricultural production. With continuous in-depth research into the mechanisms of symbiotic nitrogen fixation and technological innovations, there is potential to apply this technology to a wider range of crop species and agricultural production models [6]. This will contribute to enhancing the productivity of agricultural operations, reducing production expenses, mitigating environmental pollution, and promoting the development of agriculture toward a greener and more sustainable direction.

The establishment of SNF systems between legumes and soil rhizobia requires precise interaction between the two genomic backgrounds [7,8]. When soil rhizobia encounter phenolic compounds secreted by legume roots, they will respond to these signals by synthesizing and releasing nodulation factor (NF), surface polysaccharides, secreted proteins, and other host-specific determinants [9,10]. These rhizobial signal molecules are subsequently recognized by the root hair cells of legumes, triggering a series of host responses [11]. At the outset of nodule development, legumes need to recognize and accommodate specific rhizobia, and this process involves the initial recognition mechanisms of the plant defense system [12]. Under the action of the NFs of compatible rhizobia, the root hairs of host plants curl to form a sheath, providing conditions for rhizobia invasion. Rhizobia infiltrate the root hair sheath via root hair formation, preparing for further infection. Within the root hair sheath, rhizobia stimulate the development of a tube-like structure referred to as the infection thread (IT) [13,14,15]. The IT elongates through the root hair sheath towards the root’s cortical cells, providing a pathway for deeper rhizobial infection [16,17]. Once rhizobia successfully invade, the plant immune system needs to be moderately suppressed to allow rhizobia to proliferate within the plant and form nodules [12,18]. The plant immune system not only plays an important role in nodule formation but also finely regulates nodule development by modulating the activity of immune cells and the transmission of signaling molecules [19], which helps ensure stable reproduction of rhizobia within the plant and the normal development of nodules.

*Lotus japonicus* is a perennial herb belonging to the genus *Lotus* in the Fabaceae family. It possesses nitrogen-fixing capabilities, which make it an important model for studying plant–microbe symbiotic relationships [20]. As a model legume, *L. japonicus* not only has a small genome with a simple structure but also has a short lifecycle, which can improve the research efficiency of scientists [21]. At present, research on *L. japonicus* has mainly focused on the molecular biological characteristics, genetic improvements, and mechanisms of symbiotic nitrogen fixation. These studies offer strong support for agriculture, animal husbandry, and ecological environmental protection [22].

Bax inhibitor-1 (BI-1) is a highly conserved endoplasmic-reticulum-resident protein that acts as a cell death suppressor [23,24] and can suppress programmed cell death (PCD) in response to biotic and abiotic stresses in plants [25,26], such as those from drought [27], high salinity [27,28], or pathogen infection [29]. *BI-1* not only participates in plant stress tolerance but also plays critical roles in the growth and development of plants, such as can be seen in research showing that overexpressing *BI-1* in *Arabidopsis thaliana* may lead to growth retardation and changes in leaf morphology [30,31]. Currently, studies have shown the dual role of *BI-1* in the symbiotic relationship between legumes and rhizobia. Research indicates that significant changes in *BI-1* expression levels occur during the nascent stages of the symbiotic relationship between legumes and rhizobia, similar to changes in the expression of certain defense-related proteins [32,33].

In this report, we identified and characterized six *L. japonicus* BI-1 family genes. The potential functions of these *L. japonicus BI-1* genes in nodulation or nodule formation were explored by using expression profiles among different roots. The function analysis of *LjBI-1a* (*Lj0g3v0072129*) revealed that *LjBI-1a* promotes nodulation and nodule formation. These studies provide valuable insights for the exploration of the symbiotic roles of *L. japonicus BI-1* genes.

## 2. Materials and Methods

### 2.1. Identification of the LjBI-1 Gene Family

To identify the genes belonging to the Bax inhibitor-1 family in *L. japonicus*, we searched the *L. japonicus* database (https://www.kazusa.or.jp/lotus/) accessed on 6 July 2024 using the *GmBI-1a* gene sequence as the template sequence and identified six candidate *BI-1* genes. The protein sequences of these six genes were downloaded and validated by searching the NCBI (https://www.ncbi.nlm.nih.gov/) accessed on 6 July 2024 and Phytozome databases (https://phytozome-next.jgi.doe.gov/) accessed on 6 July 2024. SignalP—4.1 (https://services.Healthtech.dtu.Dk/services/SignalP-4.1/) accessed on 6 July 2024 and ExPasy (https://web.expasy.org/protparam) accessed on 6 July 2024 were used to analyze the protein signal peptide, molecular weight, amino acid number, isoelectric point, and other information of these six *BI-1* genes.

### 2.2. Phylogenetic Relationship Analysis and Amino Acid Sequence Analysis

Using Clustal W, a multiple sequence alignment tool, a comparative analysis was conducted on BI-1 proteins from *L. japonicus*, *Oryza sativa*, *Glycine max*, *A. thaliana*, and *Medicago truncatula*. Following this alignment, a neighbor-joining phylogenetic analysis was performed using MEGAX64 [34], with a Bootstrap value set at 1000 to ensure the robustness and reliability of the resulting phylogenetic tree. Lastly, the phylogenetic tree was visually enhanced and improved using the Evolview online tool accessible at (https://evolgenius.info/evolview-v2/#login) accessed on 7 July 2024, providing a more intuitive and aesthetically pleasing representation of the evolutionary relationships among the species.

The conserved amino acid sequences of the six *LjBI-1* genes were aligned using the NCBI Online Comparison Tool. Three conserved motifs were identified, including the Bax inhibitor (BI)-1 protein family motif, the Integral membrane protein YbhL motif, and the Conjugal transfer coupling protein TraG motif.

### 2.3. Plant Materials and Growth Conditions

The wild-type or transgenic *L. japonicus* ‘MG-20’ seedlings were grown in a chamber under a 16 h light/8 h dark cycle at 22 °C. Following a week of acclimatization, the plants were inoculated with the *M. loti* strain MAFF303099 and cultivated in the same medium without ammonium nitrate.

### 2.4. Generation of Transgenic Hairy Roots

The hairy root transformation method of *L. japonicus* currently used in research has the characteristics of high efficiency, simple cultivation conditions, and strong genetic stability, and has important value in plant gene function research and genetic analysis [35]. The ‘MG-20’ wild-type *L. japonicus* was employed to generate transgenic hairy roots via a process mediated by *A. rhizogenes*, a method documented in previous studies [34,35]. Aseptically cultivated seedlings on agar were severed at their hypocotyl bases and marinated in a broth of *A. rhizogenes* LBA1334 carrying plasmids for a 30 min duration within a Petri dish. Subsequently, cotyledon-attached seedlings were positioned on MS medium agar plates fortified with 1.5% (*w*/*v*) sucrose and nurtured in a growth chamber for a span of five days. Following this, they were relocated to agar plates infused with 250 μg/mL cefotaxime to develop further for an additional ten days, promoting the proliferation of hairy roots from the hypocotyl base. A short tip (2 to 3 mm) from each hairy root was excised and subjected to a GUS activity test in a staining solution (including 1 mm K3Fe(CN)6, 1 mm K4Fe(CN)6, 10 mm Na2EDTA, 0.1% (*w*/*v*) N-laurylsarcosine, 0.1% (*v*/*v*) Triton X-100, and 100 mm sodium phosphate buffer at pH 7.0) at 37 °C in the dark overnight. Each hairy root was marked, and the tips of hairy roots that did not exhibit GUS activity were excised, whereas those that displayed GUS activity were preserved to continue their growth. Each seedling was permitted to retain one or two transgenic hairy roots. The seedlings equipped with transgenic hairy roots were then transferred to pots containing a blend of vermiculite and sand in equal proportions with a half-strength Broughton and Dilworth (B&D) medium [36]. They were cultivated in a chamber under the conditions of a 16 h light/8 h dark cycle at a temperature of 22 °C. Following a week of acclimatization, the plants were inoculated with the *M. loti* strain MAFF303099 and grown in the same medium without ammonium nitrate.

### 2.5. LjBI-1-Specific RNAi

Two specific RNAi constructs were designed for *LjBI-1a*, aiming to silence a 135 bp sequence within the 5′-untranslated region (labeled as RNAi-1) and a 199 bp sequence within the 3′-untranslated region (labeled as RNAi-2), respectively. For comparison, control hairy roots were produced using the cloning vector CAM-BIA1301-35S-int-T7. Transgenic hairy roots were inoculated with *M. loti* strain MAFF303099 for nodulation, and nodulation phenotypes were scored after 4 weeks.

### 2.6. qPCR Analysis

Total RNA was extracted using TRIpure reagent (Aidlab, Beijing, China) according to the manufacturer’s guidelines. The quality and quantity of the RNA were checked using agarose gel electrophoresis and a nanophotometer. First-strand cDNA was synthesized using HiScript IV RT SuperMix for qPCR (+gDNA wiper) from Vazyme (Nanjing, China). The specific primers for the tested genes were synthesized by tsingke Biotech (Beijing, China). and are listed in Table S1. The qPCR reactions were conducted in a 20 μL volume, with a cycling protocol that included initial denaturation at 95 °C for 5 min, followed by 39 cycles of 95 °C for 10 s, 60 °C for 10 s, and 72 °C for 20 s, and were run on a Bio-Rad CFX96 real-time PCR system, using iTaq Universal SYBR Green Supermix from Bio-Rad (Hercules, CA, USA). The relative expression levels of the tested genes were analyzed using the 2^−∆∆Ct^ method, and *Lj-Actin7* (*Lj1g0015665*) was selected as the internal control gene to normalize the expression levels of the tested genes. Additionally, all of the qPCR analyses were repeated over three times.

### 2.7. Statistical Analysis

The relative experiments were repeated more than three times. The presented values are the means ± SDs. Asterisks represent significant differences, as determined by Student’s *t*-test (** *p* < 0.01, * 0.01 < *p* < 0.05, ns: *p* > 0.05).

### 2.8. Rhizobial Infection Assay

For the rhizobial infection experiment, the transgenic hairy roots were infected with the *M. loti* strain MAFF303099, which stably expresses the lacZ reporter gene, and were cultivated in pots filled with a 1:1 mixture of sand and vermiculite. Ten days post-inoculation, the transgenic hairy roots were subjected to β-galactosidase activity staining based on the protocols outlined in previous studies [37,38] and then examined under bright-field illumination using an Olympus BX51 microscope.

## 3. Results

### 3.1. Genome-Wide Identification of LjBI-1 Genes in L. japonicus

To identify the *BI-1* genes in *L. japonicus*, we searched the *L. japonicus* database (https://www.kazusa.or.jp/lotus/) accessed on 6 July 2024, using the *GmBI-1a* gene sequence as the template sequence, and identified six candidate *BI-1* genes; then, these genes were validated by searching the NCBI and the Phytozome databases (*L. japonicus* Lj1.0v1). These genes were named *LjBI-1a~LjBI-1f* based on their positions in chromosomes. Detailed information is listed in Table 1. These identified *L. japonicus BI-1* genes encode peptides with 240 (*LjBI-1b*) ~255 amino acid residues (*LjBI-1f*), an isoelectric point (pI) of 6.41 (*LjBI-1f*) ~9.26 (*LjBI-1c*), and a proportion of SignalIP of 0.021% *(LjBI-1a*) ~0.228% (*LjBI-1f*). The exon and intron structures of the six *LjBI-1* genes were analyzed by comparing their cDNA sequences with their genomic sequences. The results showed that most of the *LjBI-1* family genes contained four exons and three introns; only *LjBI-1c* contained six exons and five introns. It was found that only *LjBI-1c* has two transcripts; the rest of the genes have only one transcript.

### 3.2. Phylogenetic Analysis of BI-1 Genes from L. japonicus, M. truncatula, G. max, A. thaliana, and O. sativa

**Figure 1:**
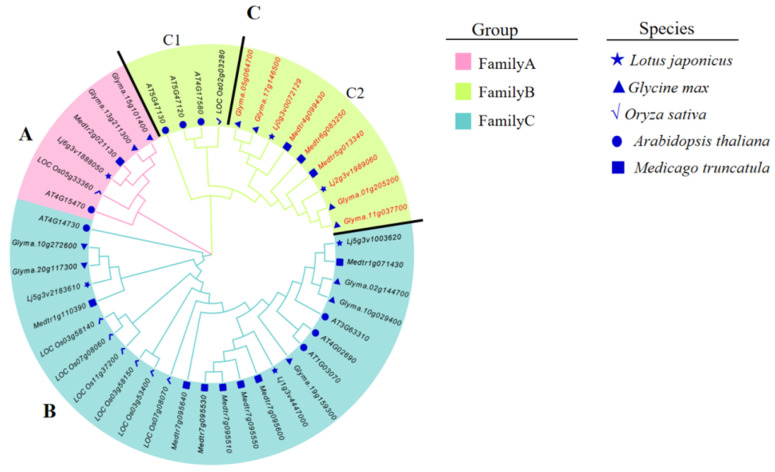
Phylogenetic analysis of the *BI-1* gene family in *L. japonicus*, *Glycine max*, *Oryza sativa*, *Arabidopsis thaliana*, and *Medicago truncatula*. The neighbor-joining phylogenetic tree was constructed using MEGA version 11.0 with a JTT + G model and 1000 bootstrap replicates. The tree divided these BI-1 proteins into three major groups (Group A to Group C) with different colors. The green, pink, and cyan colors represent the A–C groups, respectively. The different shapes in blue indicate different species. The red font represents subgroup C1: 9 legume plant *BI-1* genes.

**Figure 2:**
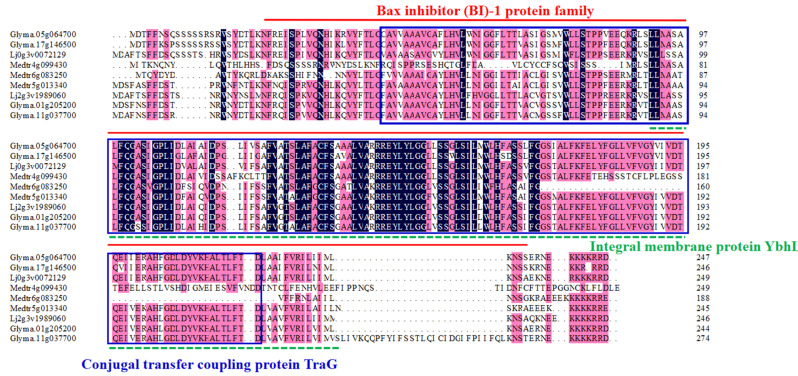
Alignments of the conserved motifs of the 9 *BI-1* genes in subgroup C1. The red, green, and blue boxes are the main retention patterns. The red line represents the Bax inhibitor (BI)-1 protein family motif; the green dashed line represents the Integral membrane protein YbhL motif; and within the blue box is the Conjugal transfer coupling protein TraG motif.

To study the phylogenetic relationships of *BI-1* genes in *L. japonicus*, *M. truncatula*, *G. max*, *A. thaliana*, and *O. sativa*, we performed a phylogenetic analysis based on alignments of the 53 full-length BI-1 protein sequences, with the results shown in Figure 1. The neighbor-joining phylogenetic tree constructed using MEGA version 11.0 divided these BI-1 proteins into three major groups (Group A to Group C), and each group featured its own distinct and highly conserved amino acid sequences (Figures S1–S3). Among them, Group A was identified as the smallest group, composed of six *BI-1* genes. Group B was recognized as the largest group, composed of six *O. sativa BI-1* genes, four *A. thaliana BI-1* genes, five *G. max BI-1* genes, seven *M. tarantula* BI-1 genes, and three *L. japonicus BI-1* genes. Group C was formed by 13 *BI-1* genes, including a specific sub branch of legumes consisting of 9 legume plant *BI-1* genes (subgroup C1, Figure 1). This result indicates that the potential biological functions of some *BI-1* genes are conserved in legume plants. We further conducted a sequence alignment analysis of these nine *BI-1* genes in subgroup C1, and the results demonstrated that these deduced peptides, containing a Bax inhibitor (BI)-1 protein family motif, an Integral membrane protein YbhL motif, and a Conjugal transfer coupling protein TraG motif [39], represented potential symbiosis-related BI-1 proteins (Figure 2).

### 3.3. Expression Profile of LjBI-1 Genes in L. japonicus Roots with and Without Inoculation of MAFF303099

**Figure 3:**
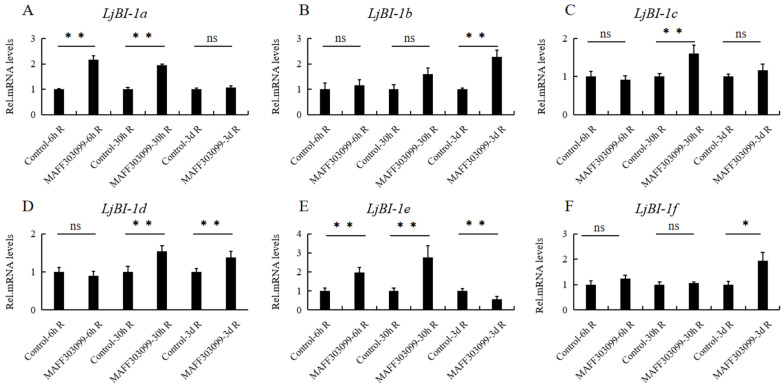
Expression profile of *LjBI-1* genes in *L. japonicus* roots with and without Rhizobium MAFF303099 inoculation at three post-inoculation time points. (**A**) *LjBI-1a* (**B**) *LjBI-1b* (**C**) *LjBI-1c* (**D**) *LjBI-1d* (**E**) *LjBI-1e* (**F**) *LjBI-1f*. The control (un-inoculated) and inoculated roots at 6 h, 30 h, and 3 d post-inoculation were used to extract RNA, and the specific primers of the six *LjBI-1* genes were utilized to perform qPCR. The expression levels of 6 *LjBI-1* genes were detected using three biological replicate samples. qPCR was used to obtain the relative expression levels of each *LjBI-1* gene, which were then normalized to the average expression level of the *L. japonicus* reference gene *QACT*. The expression levels in un-inoculated roots at the same time point were used as the controls for calculation. These results represent the mean ± SD of three independent biological repetitions. Asterisks represent significant differences, as determined by Student’s *t*-test (** *p* < 0.01; * 0.01 < *p* < 0.05; ns: *p* > 0.05).

To determine whether *LjBI-1* genes participate in symbiotic signal transduction and nodule formation, we conducted qPCR analysis to compare the expression levels of the six *LjBI-1* genes in different roots (the control and those inoculated with MAFF303099) at 6 h, 30 h, and 3 days post-inoculation (Figure 3). The inoculation of MAFF303099 significantly increased the expression of *LjBI-1a* (*Lj0g3v0072129*) and *LjBI-1e* (*Lj5g3v2183610*) (>2 fold) at 6 hR and 30 hR post-inoculation (Figure 3A,E). In the roots, at 3 days after inoculation with MAFF303099, the expression levels of *LjBI-1b* (*Lj1g3v4447000*) and *LjBI-1f* (*Lj6g3v1888050*) significantly increased (>2 fold) (Figure 3B,F). No change was observed in the expression levels of *LjBI-1c* (*Lj2g3v1989060*) and *LjBI-1d* (*Lj5g3v1003620*) (Figure 3C,D).

### 3.4. Suppression of Nodulation by LjBI-1a RNAi

**Figure 4:**
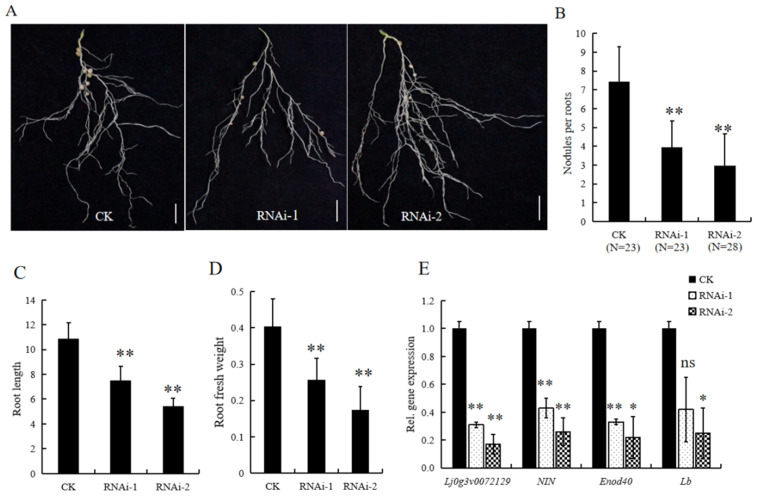
Nodulation phenotype of *LjBI-1a* RNAi in *L. japonicus*. (**A**) Photographed images representing the hairy root systems expressing the empty vector (control), *LjBI-1a* RNAi-1, and *LjBI-1a* RNAi-2. Photographs were taken 30 days after inoculation with *M. loti* MAFF303099, and plants were grown without nitrogen fertilizer. Bars = 10 mm. Mean number of nodules (**B**), mean root length (**C**), and mean root fresh weight (**D**) per plant with standard deviation (SD) of *L. japonicus* expressing the empty vector (control), *LjBI-1a* RNAi-1, and *LjBI-1a* RNAi-2 at 30 days post-inoculation with *M. loti*. (**E**) qPCR analysis of transcript levels of *LjBI-1a*, *NIN*, *Enod40*, and *Lb* in the control, *LjBI-1a* RNAi-1, and *LjBI-1a* RNAi-2 hairy roots. These results represent the mean ± SD of three independent biological repetitions. Asterisks represent significant differences, as determined by Student’s *t*-test (** *p* < 0.01; * 0.01 < *p* < 0.05; ns: *p* > 0.05).

As described above, both *LjBI-1a* (*Lj0g3v0072129*) and *LjBI-1c* (*Lj2g3v1989060*) were in the specific sub branch of legumes (Figure 1), and the expression of *LjBI-1a* was inducted by rhizobial inoculation (Figure 3A), suggesting that *LjBI-1a* may participate in nodulation. To validate this tentative result, two *LjBI-1a*-specific RNAi constructs were prepared to target a 135 bp fragment containing the 5′-untranslated region (RNAi-1) and a 199 bp fragment containing the 3′-untranslated region (RNAi-2), respectively. Control and transgenic hairy roots were inoculated with the *M. loti* strain MAFF303099 for nodulation. The cloning vector CAMBIA1301-35S-int-T7 was used to generate control hairy roots. In the results of qPCR analysis, the *LjBI-1a* transcript was reduced to 60% in RNAi-1 and 80% in RNAi-2, as compared with that in the control hairy roots (Figure 4E). At 30 days after rhizobial inoculation, the nodulation phenotypes were scored, and the results are shown in Figure 4A–D. Nodule numbers (Figure 4A,B), root length (Figure 4C), and root fresh weight (Figure 4D) in *LjBI-1a* RNAi hairy roots were significantly lower than those in the control. The expressions of two early nodulin genes, *NIN* and *ENOD40* [40,41,42], as well as a typical nodulin gene, *Lb* (leghemoglobin) [43], were decreased in *LjBI-1a* RNAi hairy roots, as compared to those in the control hairy roots (Figure 4C). These results suggest that *LjBI-1a* acts as a positive regulator in the nodulation of *L. japonicus*.

### 3.5. Suppression of Rhizobial Infection by LjBI-1a RNAi

**Figure 5:**
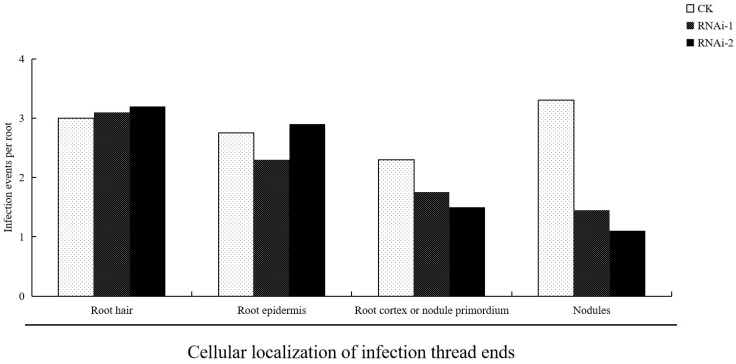
IT numbers at each infection stage in hairy roots ten days after Rhizobium inoculation. Empty vector served as control. Each root system was analyzed using twenty roots with lengths of 4–6 cm.

To study rhizobial infection in *LjBI-1a* RNAi hairy roots, we divided the infection threads [30] into four groups based on the locations of the cells at their growing tips: (1) from curled root hairs, (2) from elongated root hairs to the root epidermis, (3) through the root cortex to the nodule primordium, and (4) nodules [33]. A lacZ-labeled strain of *M. loti* [38] was used to inoculate the *LjBI-1a* RNAi hairy roots of *L. japonicus*, and the IT numbers were recorded 10 days after inoculation (Figure 5). In *LjBI-1a* RNAi hairy roots, the average number of ITs emerging from (3) and (4) was decreased compared to those in the control, and the average number of ITs emerging from (1) and (2) did not change. These data indicate that *LjBI-1a* plays a positive role in rhizobial infection in the earlier stages of nodulation.

## 4. Discussion

The SNF system between legumes and soil rhizobia provides an environmentally friendly and sustainable source of nitrogen fertilizer, reducing dependence on industrial nitrogen fertilizers and environmental pollution, and helps drive agriculture toward a green and sustainable direction [44,45]. The balanced regulation of innate immunity plays an important role in the establishment and maintenance of an SNF system [46]. BI-1, an evolutionarily conserved cell death suppressor, participates in balancing immunity and symbiosis [47], while research on the symbiotic function of the BI-1 family genes in *L. japonicus* is still lacking. In this report, we identified and characterized *L. japonicus BI-1* genes and, firstly, systematically studied the *L. japonicus* BI-1 family genes in nodulation. We also analyzed the expression patterns of these *LjBI-1* genes in roots with and without inoculation of MAFF303099, and the results provided insights into the putative roles of these *LjBI-1* genes in nodulation and nodule formation. The symbiotic function analysis of *LjBI-1a* provides useful genetic resources for improving the symbiotic nitrogen fixation ability of legumes.

BI-1 is an important apoptosis inhibitor gene that has gradually garnered attention for its role in regulating cellular apoptosis since its cloning and identification in 1998 [23]. BI-1 family genes play critical roles in cellular apoptosis regulation and plant stress tolerance, tumorigenesis, and development, as well as biological evolution and conservatism [26,30,32,47]. Despite the wide recognition of the importance of BI-1 family genes in both animals and plants, there are still relatively few studies on BI-1 family genome-wide analysis in plants [48]. This may be due to the complexity of BI-1 family genes and the differences in BI-1 genes among different species. In the present report, the entire *LjBI-1* gene family was first identified and characterized in the *L. japonicus* genome. The peptides encoded by the 6 *L. japonicus BI-1* genes contain 240–255 amino acid units, have different isoelectric points (pIs), and possess unique structural properties (Table 1). As described in the phylogenetic analysis, all three groups contain BI-1 proteins from legume plants and non-legume plants. Group C1 *BI-1* genes are in a specific sub branch of legumes (Figure 1), and they have the Bax inhibitor (BI)-1 protein family motif, Integral membrane protein YbHL motif, and Conjugal transfer coupling protein TraG motif (Figure 2). These results suggest that the *BI-1* genes in this special branch may be specific genes for legume nodulation and nitrogen fixation.

Previous research has indicated that *BI-1* genes could participate in rhizobial infection and nodule development [32,33]. However, in *L. japonicus*, the specific *BI-1* genes involved in nodulation and nodule development remain largely unknown. In the present work, we first comprehensively analyzed the expression profiles of *LjBI-1* genes in inoculated and un-inoculated roots, and the results showed that the expressions of four genes (*LjBI-1a*, *LjBI-1b*, *LjBI-1e*, and *LjBI-1f*) were induced by rhizobial inoculation (Figure 3). These results are similar to *PvBI-1a* in *Phaseolus vulgaris* roots [32] and *GmBI-1α* in soybean roots [33], suggesting that these *LjBI-1* genes may play roles in nodulation. Among the four above-mentioned *LjBI-1* genes, *LjBI-1a* is also in the specific sub branch of legumes (Figure 1). We then down-regulated the transcripts of *LjBI-1a* by RNAi and observed that *LjBI-1a* promotes nodulation and nodule formation (Figure 4 and Figure 5), similar to *PvBI-1a* [32] and *GmBI-1α* [33]. The roles of *LjBI-1a* in nodule senescence and plant immunity and the regulation mechanism of *LjBI-1a* in RNS need further in-depth research.

## 5. Conclusions

In summary, six *BI-1* family genes were identified in *L. japonicus*. The special characteristics of *LjBI-1* genes mainly reflected in their gene sequences, pI values, protein structures, and phylogenetic analysis. The expression patterns of *LjBI-1* genes in roots with and without inoculation of MAFF303099 indicated that four *LjBI-1* genes might participate in nodulation. The nodulation experiment revealed that the candidate gene *LjBI-1a* is likely to act as a positive regulatory factor in nodulation and nodule formation. These findings provide new ideas for studying the symbiotic function of all *L. japonicus BI-1* family genes, and provide useful genetic resources for improving the symbiotic nitrogen fixation ability of legumes.

## Figures and Tables

**Table 1:** Detailed information on *Lotus japonicus* Bax inhibitor-1 (BI-1) family genes.

Name	Gene ID	Chromosome Location	Transcript Numbers	Protein Size (aa)	MW (kDa)	PI	Signal Peptide	Exon	Intron
*LjBI-1a*	Lj0g3v0072129	chr4:34395727-34398462	1	249	27651.19	8.85	0.0210%	4	3
*LjBI-1b*	Lj1g3v4447000	chr1:10917466-10919589	1	240	26873.15	8.90	0.1240%	4	3
*LjBI-1c*	Lj2g3v1989060	chr2:1184961-1187596	2	246	27291.95	9.26	0.0390%	6	5
*LjBI-1d*	Lj5g3v1003620	chr5:24312170-24315258	1	242	27075.23	6.55	0.1410%	4	3
*LjBI-1e*	Lj5g3v2183610	chr5:2502444-2504025	1	240	27101.31	7.65	0.0210%	4	3
*LjBI-1f*	Lj6g3v1888050	chr6:52345422-52347607	1	255	28373.34	6.41	0.2280%	4	3

## Data Availability

The original contributions presented in the study are included in the article/Supplementary Material, further inquiries can be directed to the corresponding author.

## Supplementary Materials

The following supporting information can be downloaded at: https://www.mdpi.com/article/10.3390/genes16010058/s1. Figure S1. Alignments of conserved motifs of the 6 BI-1 genes in subgroup A; Figure S2. Alignments of conserved motifs of the 25 BI-1 genes in subgroup B; Figure S3. Alignments of conserved motifs of the 13 BI-1 genes in subgroup C; Table S1. Primers used in this study.
