# Supplementary material for: Suppression of Nodule Formation by RNAi Knock-Down of Bax inhibitor-1a in Lotus japonicus
Source: Genes (Basel). 2025 Jan 6;16(1):58. doi: 10.3390/genes16010058 (PMC11764830; doi:10.3390/genes16010058)
Supplement: Supplementary file 1 [file genes-16-00058-s001.zip › genes-3353924-supplementary.pdf]

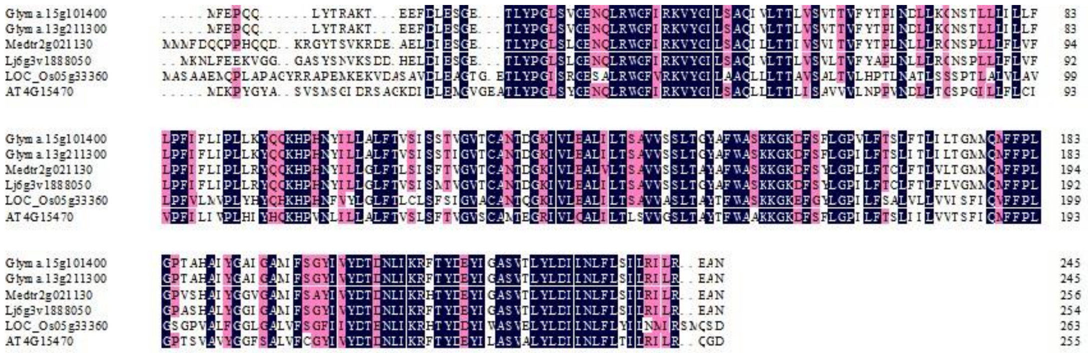

Figure S1. Alignments of conserved motifs of the 6 *BI-1* genes in subgroup A.

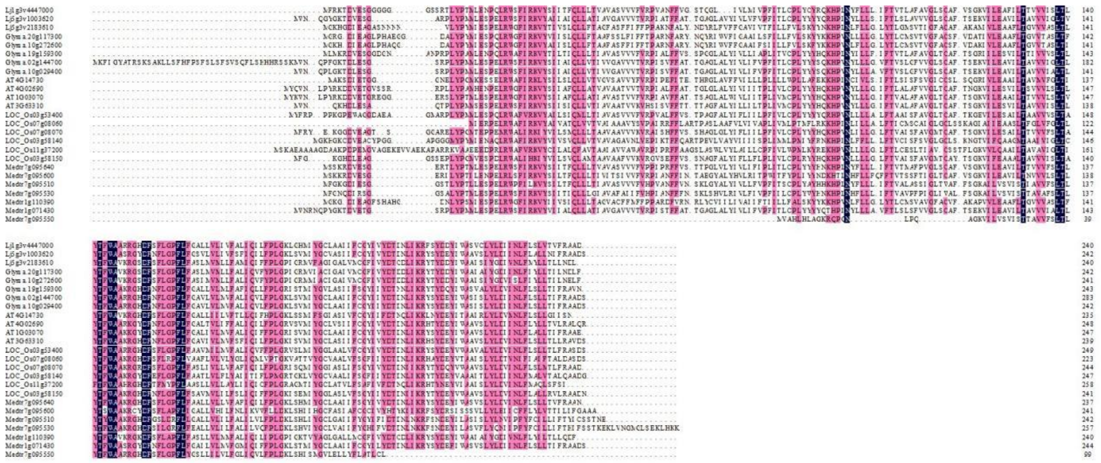

Figure S2. Alignments of conserved motifs of the 25 *BI-1* genes in subgroup B.

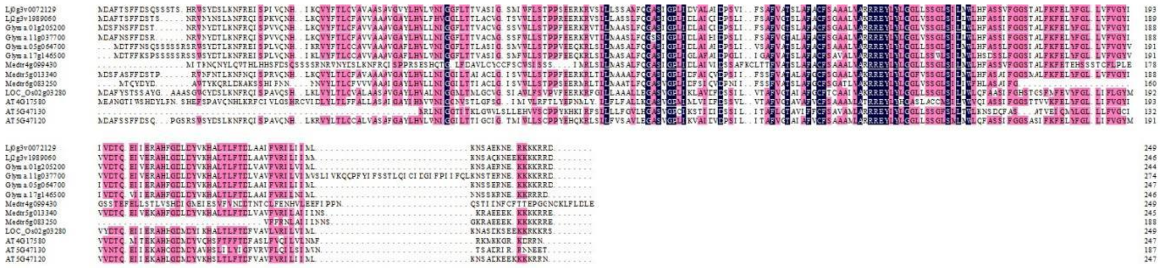

Figure S3. Alignments of conserved motifs of the 13 *BI-1* genes in subgroup C.

Table S1. Primers used in this study.

Primer name	Sequence of primer（5′ -3′ ）
F-Gm.17g146500	AGTCCCCATCGTCTTC
R-Gm.17g146500	CATGAAGGAAGGCTCC
F-NIN	AACTCACTGGAAACAGGTGCTTTC
R-NIN	CTATTGCGGAATGTATTAGCTAGA
F-ENOD40-1	GGAGGTATGCTCAAACATTC
R-ENOD40-1	GTAACTTCTCAAGAGAAGACC
F-ENOD40-2	CAAAACTCGTTATGTTGCGG
R-ENOD40-2	CACCTCAAAGGAAGAAGAACA
F-UBI	TTCACCTTGTGCTCCGTCTTC
R-UBI	AACAACAGCACACACAGACAATC
qPCR-LjBI1-1-F	GGCTTGAGCTTCTCTTCTGTAAT
qPCR-LjBI1-1-R	GAAGCTCAACTGCCTATCTAC
qPCR-LjBI1-2-F	CGGATACACGTTGGAACGGAAG
qPCR-LjBI1-2-R	GTAAGGAATATATGCAGCTGC
qPCR-LjBI1-3-F	GTTCCATGCTTTCTCCATGAC
qPCR-LjBI1-3-R	CTGCGTTTAATATGCAGGGTA
qPCR-LjBI1-4-F	GTCCTGTGGATTATTGTCGAAGC
qPCR-LjBI1-4-R	CCTACCAGGTTCATTTCAGTCTC
qPCR-LjBI1-5-F	TTCCCTCTTGGACCAATCGGG
qPCR-LjBI1-5-R	CCATAGATGGAGATAGCAGCCC
qPCR-LjBI1-6-F	CGTAGCTTATCATACTATCACCC
qPCR-LjBI1-6-R	GTATTACAGCTACCATCCGC
F-Actin-Lj	GCCATGGAGAAGAGGTGAGA
R-Actin-Lj	GGGGAAATGGAGATGAAAT
